# Reducing Aspiration Pneumonia Risk for Older People: Effect of Evidence-Based Oral Care

**DOI:** 10.1093/geroni/igab046.1408

**Published:** 2021-12-17

**Authors:** Lynette Goldberg, Leonard Crocombe, Silvana Bettiol, Anna King, Sangeeta Khadka

**Affiliations:** 1 University of Tasmania, Hobart, Tasmania, Australia; 2 Latrobe University, Latrobe University Melbourne, Victoria, Australia; 3 University of Tasmania, University of Tasmania, Hobart, Tasmania, Australia; 4 University of Tasmania, University of Tasmania, Tasmania, Australia

## Abstract

Poor oral health increases the risk of aspiration pneumonia for older people. This is due primarily to six pathogens found in the mouth: five bacteria and one fungus. With a cohort of older people who were dependent on others for their oral care, we analyzed the load and type of bacteria and fungi from swabs of cheek, gum, and tongue mucosa. There were no significant differences between the three sites for load of bacteria (H (2) = .89; p = .64); there were significant differences between the sites for type of bacteria (F (2,78) = 11.97; p <.001) with the tongue showing the greatest diversity. There were no significant differences between the three sites for load (H (2) = 2.94; p = .23) or type (F (2,77) = .46; p = .63) of fungi. We then investigated the effect of regular compared to evidence-based oral care over a six-week period, and whether evidence-based oral care could significantly reduce the absolute count of the six oral pathogens specifically related to aspiration pneumonia. Participants self-selected into Regular Care (n = 10) and Evidence-based Care (n = 17) Groups. Evidence-based oral care resulted in significant decreases (p = .02 to p < .001) in the load of four potentially pathogenic bacterial species, including E. coli, gut-based bacteria, and in an increased load of Lactobacillus reuteri, a host-protective normal flora in the mouth, compared to baseline. There were no significant differences between groups for the abundance and type of fungi.

